# Transcriptome-wide 1-methyladenosine functional profiling of messenger RNA and long non-coding RNA in bladder cancer

**DOI:** 10.3389/fgene.2024.1333931

**Published:** 2024-02-28

**Authors:** Jian-jian Yin, Yan-liang Song, Yu-feng Guo, Yuan-heng Dai, Qi Chang, Tao Wang, Guo-qiang Sun, Ping Lu, Dong-kui Song, Li-rong Zhang

**Affiliations:** ^1^ Department of Pharmacology, School of Basic Medical Sciences, Academy of Medical Sciences, Zhengzhou University, Zhengzhou, Henan, China; ^2^ College of Public Health, First Affiliated Hospital of Zhengzhou University, Zhengzhou University, Zhengzhou, Henan, China; ^3^ Department of Urology, First Affiliated Hospital of Zhengzhou University, Zhengzhou, Henan, China; ^4^ Institute of Advanced Technology, Zhengzhou University, Zhengzhou, Henan, China; ^5^ State Key Laboratory for Esophageal Cancer Prevention and Treatment, Zhengzhou University, Zhengzhou, Henan, China

**Keywords:** bladder cancer, m^1^A, mRNA, lncRNA, tumorigenesis

## Abstract

**Introduction:** Post-transcriptional RNA modifications are crucial regulators of tumor development and progression. In many biological processes, N^1^-methyladenosine (m^1^A) plays a key role. However, little is known about the links between chemical modifications of messenger RNAs (mRNAs) and long noncoding RNAs (lncRNAs) and their function in bladder cancer (BLCA).

**Methods:** Methylated RNA immunoprecipitation sequencing and RNA sequencing were performed to profile mRNA and lncRNA m^1^A methylation and expression in BLCA cells, with or without stable knockdown of the m^1^A methyltransferase tRNA methyltransferase 61A (TRMT61A).

**Results:** The analysis of differentially methylated gene sites identified 16,941 peaks, 6,698 mRNAs, and 10,243 lncRNAs in the two groups. Gene ontology enrichment and Kyoto Encyclopedia of Genes and Genomes pathway analyses of the differentially methylated and expressed transcripts showed that m^1^A-regulated transcripts were mainly related to protein binding and signaling pathways in cancer. In addition, the differentially genes were identified that were also differentially m^1^A-modified and identified 14 mRNAs and 19 lncRNAs. Next, these mRNAs and lncRNAs were used to construct a lncRNA-microRNA-mRNA competing endogenous RNA network, which included 118 miRNAs, 15 lncRNAs, and 8 mRNAs. Finally, the m^1^A-modified transcripts, SCN2B and ENST00000536140, which are highly expressed in BLCA tissues, were associated with decreased overall patient survival.

**Discussion:** This study revealed substantially different amounts and distributions of m^1^A in BLCA after TRMT61A knockdown and predicted cellular functions in which m^1^A may be involved, providing evidence that implicates m^1^A mRNA and lncRNA epitranscriptomic regulation in BLCA tumorigenesis and progression.

## 1 Introduction

Bladder cancer (BLCA) is the most common malignant tumor of the urinary system and ranks sixth among malignancies in men (Siegel et al., 2022). The incidence of BLCA in China ranks first among male urogenital tumors and second only to prostate cancer worldwide (Sung et al., 2021; Xia et al., 2022). Due to the lack of effective diagnostic approaches, approximately 20%–30% of BLCA cases are diagnosed as muscle-invasive bladder cancer (MIBC) (Patel et al., 2020; Xia et al., 2022). Newly diagnosed cases present with non-muscle-invasive bladder cancer (NMIBC), which is usually treated by transurethral resection and intravesical therapy. However, MIBC usually relapses and progresses (Lenis et al., 2020; Patel et al., 2020). With recent developments in immunotherapy, advanced BLCA may be treated using anti-PD-1/PD-L1 therapy (Song et al., 2019). However, no effective treatment is currently available for most patients with relapsed MIBC or metastatic BLCA. Therefore, further studies to identify novel pathways involved in BLCA development and progression are required to develop more efficient therapies.

Post-transcriptional RNA modifications have emerged as additional layers of epitranscriptomic regulation (Barbieri and Kouzarides, 2020; Han et al., 2020; Haruehanroengra et al., 2020; Qu et al., 2022). Accumulating evidence suggests that RNA modifications play critical roles in various biological processes that contribute to disease pathogenesis. Currently, more than 170 modifications have been described in all classes of RNAs (Han et al., 2020), including N^6^-methyladenosine (m^6^A), N^1^-methyladenosine (m^1^A), N^7^-methylguanosine (m^7^G), and 5-methylcytosine (m^5^C) (Barbieri and Kouzarides, 2020; Han et al., 2020; Haruehanroengra et al., 2020). Among them, m^1^A is involved in stabilizing RNA structure, splicing, cell proliferation, and cell death (Dominissini et al., 2016; Li et al., 2017). M^1^A is characterized by tRNA and rRNA, which have a major influence on its structure and function owing to the presence of both methyl adducts and a positive charge under physiological conditions (Roovers et al., 2004). Although high-throughput sequencing methods have been developed for the m^1^A methylome, m^1^A has also been detected in messenger RNAs (mRNAs), mitochondrial transcripts, and noncoding RNAs (Oerum et al., 2017; Safra et al., 2017; Tang et al., 2020). M^1^A methylation involves three types of enzymes: m^1^A methyltransferases [nucleomethylin, tRNA methyltransferase 10C (TRMT10C), TRMT61B, and TRMT6/TRMT61A (Chujo and Suzuki, 2012; Li et al., 2017; Safra et al., 2017; Sharma et al., 2018)], demethylases (alkB homolog 1, histone H2A dioxygenase and alkB homolog 3, alpha-ketoglutarate dependent dioxygenase [Li et al., 2016; Woo and Chambers, 2019)], and m^1^A-binding proteins [YTH N6-methyladenosine RNA binding proteins F1–3, and YTH N6-methyladenosine RNA binding protein C1 (Dai et al., 2018; Kisan and Chhabra, 2022)].

Recent studies have revealed that alterations in m^1^A regulators are closely associated with multiple tumors (Shi et al., 2015; Macari et al., 2016; Zhao et al., 2019; Wang et al., 2020; Wang YY. et al., 2021; Zheng et al., 2021; Li et al., 2022; Mao et al., 2022). For instance, the expression of TRMT6/TRMT61A is significantly increased in hepatocellular carcinoma tissues and increased TRMT6 and TRMT61A levels are negatively associated with patient prognosis (Wang YY. et al., 2021). Our previous studies have shown that TRMT61A is highly expressed in BLCA tissues and that high TRMT61A expression is associated with a low disease-free survival rate (Shi et al., 2015). Furthermore, the knockdown of TRMT6 and TRMT61 induces glioma cell death and growth inhibition (Macari et al., 2016). However, current knowledge of m^1^A modification is mainly based on tRNA studies, and little is known about its role in mRNA and long non-coding RNA (lncRNA) modifications. The distribution and function of m^1^A across diverse classes of RNA have been explored. Post-transcriptional modifications of mRNAs and long noncoding RNAs (lncRNAs) may alter the expression and activity of mRNAs, ncRNAs, and proteins, resulting in epitranscriptomic changes in living cells (Li et al., 2016; Li et al., 2017). LncRNAs characteristically fulfill regulatory or structural roles in different biological and pathological activities and are distinct from protein-coding genes (Quinn and Chang, 2016). However, knowledge of the prevalence and transcriptome-wide distribution of m^1^A in mRNAs and lncRNAs is limited.

This study aimed to gain a deeper understanding of m^1^A methylation in BLCA mRNAs and lncRNAs. To this end, m^1^A was globally mapped using RNA methylated RNA immunoprecipitation sequencing (MeRIP-seq) in BLCA 5637 cells with or without TRMT61A knockdown and a genome-wide integrated analysis of m^1^A methylation, and the transcriptome was performed to characterize the crosstalk between m^1^A methylation and mRNA and lncRNA regulation (Supplementary Figure S1). These findings provide new insights into the m^1^A epitranscriptomic regulation of mRNA and lncRNAs in BLCA for the development of novel therapies.

## 2 Materials and methods

### 2.1 Cells

The human BLCA cell line 5637 was purchased from the Chinese Academy of Cell Resources Center (Shanghai, China). Cells were incubated in Roswell Park Memorial Institute-1640 medium (Gibco, United States) containing 10% fetal bovine serum (Corning, United States) and 1% penicillin and streptomycin (Solarbio, China) at 37°C with 5% CO_2_. The cell line was authenticated using short tandem repeat profiling and routinely tested to exclude *Mycoplasma* contamination.

### 2.2 Patients and tumor samples

Eighteen pairs of BLCA samples and adjacent tissues were collected from patients aged 52–83 years (median age of 69.5 years) after total or partial cystectomy at the First Affiliated Hospital of Zhengzhou University from September 2019 to September 2022. Of these, 4 and 14 patients were diagnosed with low- and high-grade BLCA, respectively (O’Sullivan et al., 2017). BLCA and adjacent normal tissues located 2 cm away from the cancer tissue were collected within 1 h of bladder isolation surgery. Specimens were washed with normal saline, immediately snap-frozen in liquid nitrogen within de-enzymatic cryopreservation tubes, and stored at −80°C for later use. This study was approved by the Ethics Committee of the First Affiliated Hospital of Zhengzhou University, and written informed consent was obtained from the patients or their relatives prior to the study.

### 2.3 Cell transfection

A stable knockdown plasmid of TRMT61A was generated using the “pHBLV-U6-MCS-EF1-luc-T2A-Puro” vector synthesized by Hanbio Biotechnology (Shanghai, China). To construct a stable cell line (short hairpin (sh)-TRMT61A), 293T packaging cells were used for lentiviral production. Lentiviral infection was performed according to the manufacturer’s instructions. The sh-TRMT61A oligonucleotide sequences were as follows.

Sense: GAT​CCG​GCA​CTC​AGT​TGA​CCT​TAT​TTC​AAG​AGA​ATA​AGG​TCA​ACT​GAG​TGC​CTT​TTT​TG.

Antisense: AAT​TCA​AAA​AAG​GCA​CTC​AGT​TGA​CCT​TAT​TCT​CTT​GAA​ATA​AGG​TCA​ACT​GAG​TGC​CG.

### 2.4 RNA isolation and reverse transcriptase-quantitative polymerase chain reaction (RT-qPCR)

Total RNA was extracted from 5637 cells using the TRIzol reagent (Invitrogen, United States). The RNA concentration of each sample was determined using a NanoDrop 2000 (Thermo Fisher, United States). The quality of the RNAs was subsequently determined by measuring the OD260/OD280 value, and RNA purity was confirmed by measuring the OD260/OD230 value. And the ratio of 260/280 between 1.8 and 2.0 and the ratio of 260/230 between 2.0 and 2.2 were considered to be pure RNA. Agarose gel electrophoresis was used for the integrity and quality testing of total RNA. Complementary DNA was reverse-transcribed from 1 μg of total RNA using the Prime Script RT Reagent kit (Takara Bio, Japan), according to the manufacturer’s instructions, and then used as a template for qPCR using the SYBR Green Master Mix kit (Thermo Fisher). The thermal cycling program included the following steps: 50°C for 2 min, denaturation at 95°C for 2 min, followed by 40 cycles of 95°C for 15 s and 60°C for 1 min. Primers were synthesized by Beijing Liuhe Huada Gene Technology Co. (Supplementary Table S1).

### 2.5 Western blot analysis

Cellular proteins were solubilized in radioimmunoprecipitation assay (RIPA) lysis buffer containing a protease inhibitor cocktail (MedChem Express, United States). Protein concentration was determined using the bicinchoninic acid method (Beyotime, China) and adjusted to the same concentration across groups. Proteins were separated using sodium dodecyl sulfate-polyacrylamide gel electrophoresis (Solarbio, China) and electrotransferred to polyvinylidene difluoride membranes (Millipore, United States). The membranes were immunoblotted at 4°C overnight with the following antibodies: anti-TRMT61A (1:2000; Thermo Fisher) and anti-glyceraldehyde 3 phosphate dehydrogenase (GAPDH) (1:5000; Proteintech, China). The membranes were washed with Tris-buffered saline containing 0.1% Tween^®^ 20 and incubated with alkaline phosphatase-conjugated affinipure goat anti-rabbit/mouse IgG (H + L) (1:10,000; Proteintech) for 2 h at 37°C. Results were quantitatively analyzed using AlphaView software (ProteinSimple, United States).

### 2.6 Dot blot assays

Purified RNA was denatured and spotted onto a Magna nylon transfer membrane (GE Healthcare, Chicago, IL, United States), followed by UV-crosslinking. The membranes were blocked with 5% skim milk at 37°C for 1 h and incubated with mouse monoclonal anti-m^1^A antibody (MBL, Japan), followed by horseradish peroxidase (HRP)-conjugated anti-mouse antibody. The results were quantitatively analyzed using High-sig ECL Western blotting Substrate (Amersham ImageQuant 800, Japan). Next, 0.02% methylene blue in 0.3 M sodium acetate was used to visualize the total RNA.

### 2.7 Processing and analysis of mRNA and lncRNA expression profiles

RNA sequencing (RNA-seq) data of BLCA 5637 control (sh-NC) and TRMT61A knockdown (sh-TRMT61A) 5637 cells using the Illumina HiSeq RNA-Seq platform were retrieved from the University of California Santa Cruz Xena Browser (https://xena.ucsc.edu/). A total of 38,939 mRNAs and 23,592 lncRNAs were annotated from the RNA-seq data using GENCODE (v23) annotations. Differential expression analysis was conducted using the R package “DESeq2.” LncRNAs with |log2 (fold change) | >1 and False Discovery Rate-corrected *p*-values <0.05 were identified as differentially expressed lncRNAs. Hierarchical clustering analysis of samples based on lncRNA expression was performed using the R package “heatmap” with the “ward. D2” method (Wang et al., 2010; Olarerin-George and MetaPlotR, 2017).

### 2.8 MeRIP-seq

Fragmented RNA was incubated with an anti-m^1^A polyclonal antibody (MBL, Japan) in an immunoprecipitation buffer for 2 h at 4°C. The reaction mixture was then immunoprecipitated with protein A magnetic beads (Thermo Fisher) at 4°C for 2 h. Next, the bound RNA was eluted from the beads with m^1^A antibody in IPP buffer and extracted with the TRIzol reagent. Extracted RNA was prepared using the NEB Next Ultra II Directional RNA Library Prep Kit (NEB, United States). Both the input sample (without immunoprecipitation) and the m^1^A antibody-immunoprecipitated samples were subjected to 150 bp paired-end sequencing on an Illumina HiSeq sequencer.

### 2.9 Detection of m^1^A levels using ultra-high performance liquid chromatography (UHPLC)-tandem mass spectrometry (MS/MS)

According to the experimental method established in the early stages of this research (Chang et al., 2023), the peak time of the unknown peak of the tested sample was compared with that of the nucleoside standard to identify a variety of nucleosides. The ratio of different concentrations of the nucleoside standard to the internal standard was used to establish a working curve, which was used to calculate nucleoside concentrations.

### 2.10 Sequencing data analysis

Paired-end reads were harvested from an Illumina Novaseq (6000)–(4000) sequencer and were quality-controlled using Q30. After 3’ adaptor-trimming and low-quality reads were removed using the cut-adapt software (v1.9.3), the high-quality trimmed reads were used to analyze mRNAs and ncRNAs. High-quality reads were aligned to the reference genome/transcriptome using STAR software (v2.5.1b) (Dobin et al., 2013), and mRNAs and lncRNAs were detected and identified using DCC software (v0.4.4). EdgeR (v3.16.5) was used to normalize the data and perform differentially expressed mRNA and lncRNA analyses. Gene Ontology (GO) and Kyoto Encyclopedia of Genes and Genomes (KEGG) analyses were performed for differentially expressed mRNA and lncRNA-associated genes. Methylated sites on RNAs were identified using MACS software, and the peaks were visualized using the integrative genomics viewer (IGV) software (Zhang et al., 2008; Thorvaldsdottir et al., 2013).

### 2.11 Statistical analysis

Statistical analyses were performed using GraphPad Prism (version 9.0; GraphPad, United States) and the SPSS software (version 21.0; SPSS Inc., United States). The experiments were independently repeated at least three times, and representative data are presented as the mean ± standard deviation. Qualitative data were evaluated using the Chi-square test. A two-tailed Student’s t-test was used for comparison between the two groups. *p*-values for every result are labeled in the figures, and *p* < 0.05 was considered significantly different.

## 3 Results

### 3.1 TRMT61A knockdown reduced transcriptome-wide m^1^A methylation of mRNAs and lncRNAs in human BLCA cells

**FIGURE 1:**
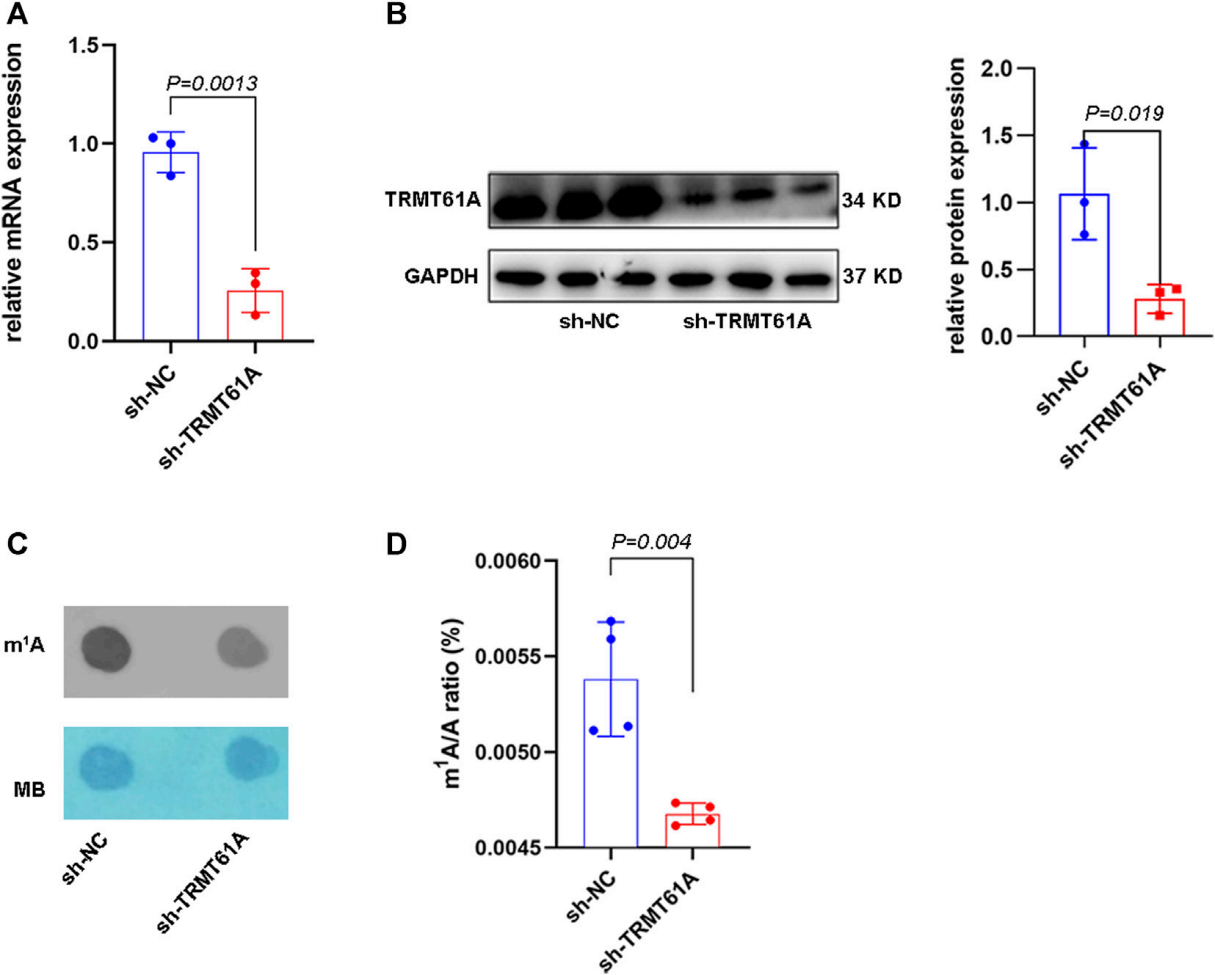
tRNA methyltransferase 61A (TRMT61A) knockdown (KD) decreases RNA N1-methyladenosine (m^1^A) modification in 5637 cells. **(A)** Reverse transcriptase-quantitative polymerase chain reaction (RT-qPCR) analysis of TRMT61A levels in short hairpin-negative control (sh-NC) and sh-TRMT61A 5637 cells (*n* = 3). **(B)** Western blot analysis of TRMT61 protein levels after TRMT61A KD in 5637 cells (n = 3). **(C)** m^1^A levels in sh-NC and sh-TRMT61A 5637 cells, as examined by RNA dot blot. Methylene blue (MB) staining serves as a loading control. **(D)** Ultra-high performance liquid chromatography-tandem mass spectrometry (UHPLC–MS/MS) assays quantify m^1^A levels in total RNA from sh-NC and sh-TRMT61A 5637 cells (*n* = 4).

A TRMT61A stable knockdown (sh-TRMT61A) 5637 cell line was generated using a lentiviral shRNA construct. Knockdown efficiency was verified using RT-qPCR and Western blot (Figures 1A, B). Moreover, dot blot and UHPLC–MS/MS assays showed that m^1^A levels in total RNA decreased after TRMT61A knockdown (Figures 1C, D). These data demonstrated that TRMT61A was required for RNA m^1^A modification.

**FIGURE 2:**
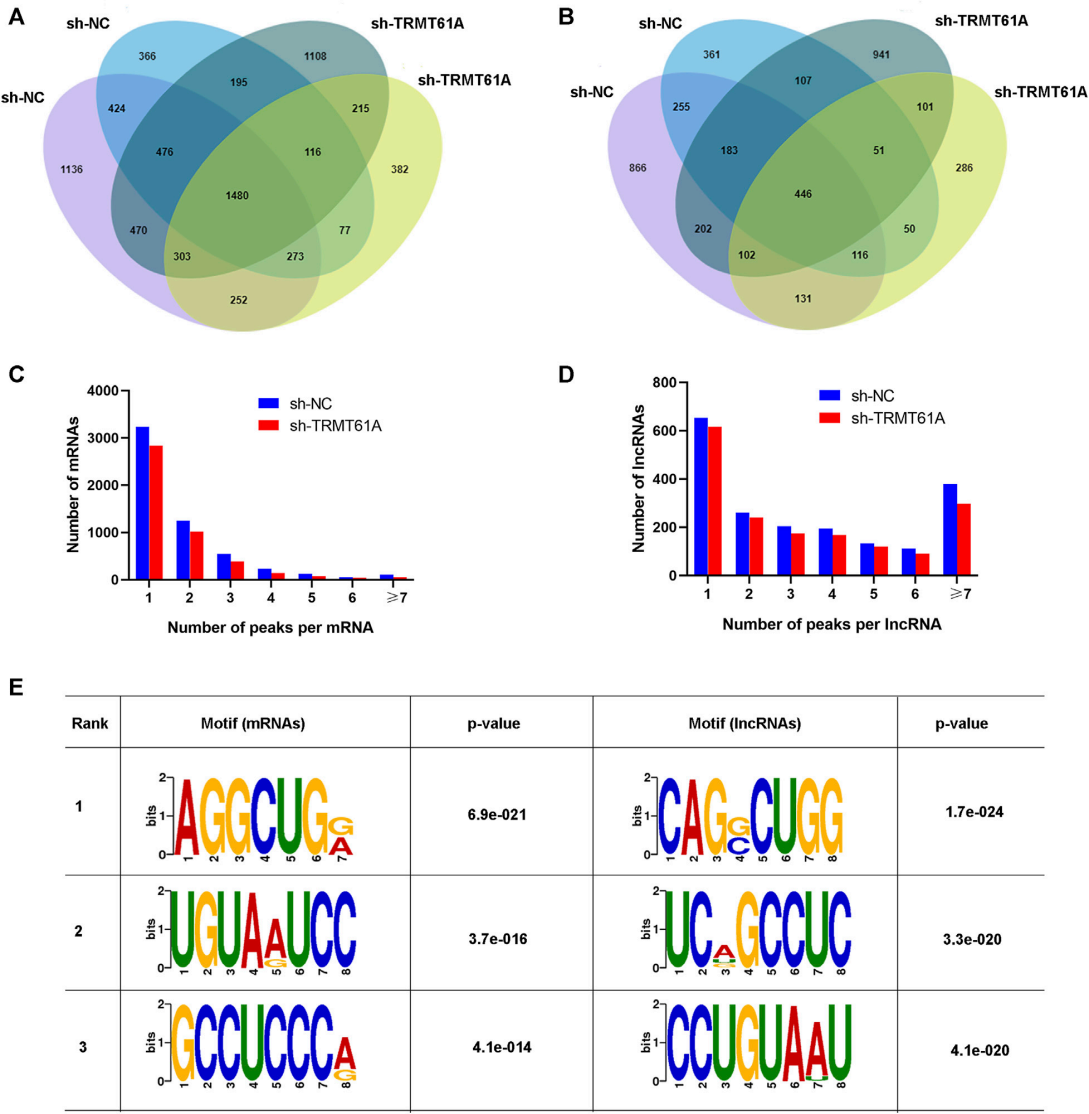
tRNA methyltransferase 61A (TRMT61A) knockdown reduces transcriptome-wide N1-methyladenosine (m^1^A) modifications in messenger RNAs (mRNAs) and long noncoding RNAs (lncRNAs) in 5637 cells. **(A, B)** Venn diagram showing the numbers of m^1^A modification sites identified in mRNAs and lncRNAs from short hairpin-negative control (sh-NC) and sh-TRMT61A 5637 cells. **(C, D)** The percentage of mRNAs and lncRNAs harboring different numbers of m^1^A peaks in sh-NC and sh-TRMT61A 5637 cells, with the majority harboring only one m^1^A peak. **(E)** The sequence motif of m^1^A sites in sh-NC and sh-TRMT61A 5637 cells and respective *p* values (the enrichment *p*-value is calculated using Fisher’s Exact Test for the enrichment of the motif in the positive sequences).

Next, RNA-seq and transcriptome analyses were performed using sh-NC and sh-TRMT61A 5637 cells. To examine transcriptome-wide RNA m^1^A methylation, MeRIP-seq of mRNAs and lncRNAs was performed. Analysis results of differential genes showed that a total of 16,941 peaks, 6,698 mRNA genes, and 10,243 lncRNAs were identified. These included 1,919 differentially expressed mRNA genes (736 upregulated and 1,183 downregulated), and 3,172 differentially expressed lncRNAs (1,157 upregulated and 2,015 downregulated). The m^1^A methylation sequencing results revealed 5,568 mRNA m^1^A peaks in sh-NC 5637 cells and 5,374 mRNA m^1^A peaks in sh-TRMT61A 5637 cells. Of these, 1,480 mRNA m^1^A peaks overlapped between sh-NC and sh-TRMT61A 5637 cells (Figure 2A and Supplementary Table S2). For lncRNAs, there were 4,285 m^1^A peaks in sh-NC 5637 cells, 3,682 m^1^A peaks in sh-TRMT61A 5637 cells, and 446 lncRNA m^1^A peaks that overlapped between sh-NC and sh-TRMT61A 5637 cells (Figure 2B and Supplementary Table S2).

Next, the abundance of m^1^A peaks in the mRNAs and lncRNAs was analyzed. All m^1^A methylated transcripts were divided into seven groups based on the number of peaks per transcript. Notably, most m^1^A-methylated mRNAs and lncRNAs contained one m^1^A site, and we also found the peaks of m^1^A modifications were reduced in both mRNAs and lncRNAs (Figures 2C, D). Interestingly, lncRNAs had a relatively high content of seven or more m^1^A peaks. To determine the presence of an m^1^A motif, the sequences of the m^1^A-methylated peaks were scanned. AGGCUG was determined to be the most reliable motif (Figure 2E). Taken together, these data demonstrated that TRMT61A was required for the m^1^A modification of both mRNAs and lncRNAs through the AGGCUG motif.

### 3.2 m^1^A localized at all chromosomes and at transcription start sites, CDSs, and 5′-untranslated regions (UTRs) of transcripts

**FIGURE 3:**
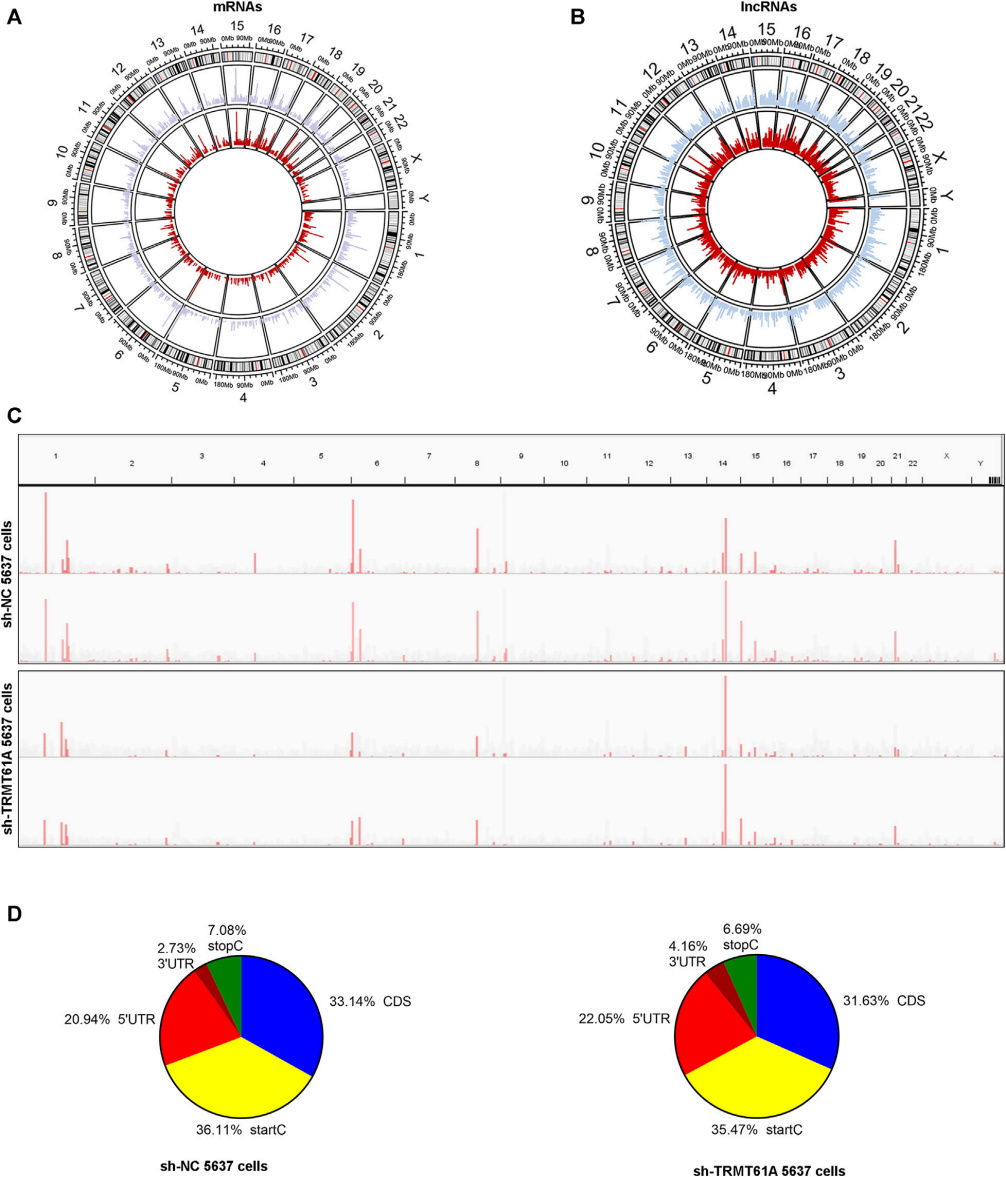
N1-methyladenosine (m^1^A) localizes at all chromosomes and at transcription start sites, CDSs, and 5′-UTRs of transcripts. **(A, B)** Circos plots showing the distribution of m^1^A methylation sites in messenger RNAs (mRNAs) and long noncoding RNAs (lncRNAs) on each chromosome. The red line represents m^1^A sites in short hairpin (sh)-TRMT61A 5637 cells and the blue line represents m^1^A sites in sh-NC 5637 cells. **(C)** Representative RNA m^1^A modification peaks in sh-NC and sh-TRMT61A 5637 cells (the horizontal axis represents human chromosomes, and the vertical axis indicates m^1^A enrichment). **(D)** Pie charts illustrate the percentage of m^1^A peaks in five non-overlapping segments of RNAs in the sh-NC cells (left pie chart) and sh-TRMT61A 5637 cells (right pie chart). Transcription start codon (StartC), 5′-untranslated region (5′-UTR), coding sequence (CDS), 3′-untranslated region (3′-UTR), and stop codon (StopC).

To determine how m^1^A modification was distributed in BLCA, the distribution of m^1^A methylation sites was investigated throughout the whole genome of sh-NC and sh-TRMT61A 5637 cells (Figures 3A, B). m^1^A sites were dispersed across all chromosomes and RNA genes undergoing m^1^A modification were scattered across all chromosomes. IGV visualization analysis was simultaneously used to visually present the sequencing results, which revealed a discernible reduction in the overall level of m^1^A modification peaks following TRMT61A knockdown (Figure 3C). To analyze the distribution profile of the m^1^A peaks within mRNAs and lncRNAs together, the peaks were categorized into five transcript segments: 5′-UTR, start codon, coding sequence (CDS), stop codon, and 3′-UTR. However, there was no difference in the sites of m^1^A modifications between sh-NC and sh-TRMT61A cells, as the percentage of m^1^A modifications at different regions of mRNAs (5′-UTR, start codon, CDS, stop C, and 3′-UTR regions) and lncRNAs remained the same with or without TRMT61A knockdown (Figure 3D). These data suggested that RNA m^1^A modifications occurred in transcripts from all chromosomes in a TRMT61A-dependent manner and that m^1^A modifications mostly localized at the transcription start site, CDS, and 5′-UTR.

### 3.3 Combined analysis of m^1^A MeRIP-seq and RNA-seq data revealed a positive correlation between m^1^A modification and gene expression

**FIGURE 4:**
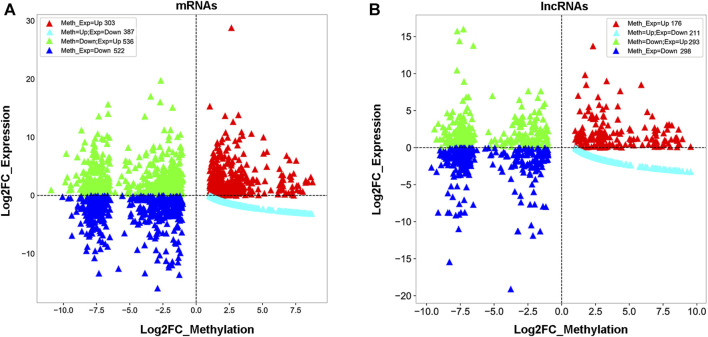
Combined analysis of N1-methyladenosine (m^1^A) methylated RNA immunoprecipitation sequencing (MeRIP-seq) and RNA sequencing data reveals a correlation between m^1^A modification and gene expression. **(A, B)** Distribution of messenger RNA (mRNA) and long noncoding RNA (lncRNA) with a significant change in both m^1^A and expression levels after stable knockdown of tRNA methyltransferase 61A (TRMT61A) in 5637 cells.

To understand whether m^1^A modification regulated gene expression in 5637 cells, a combined analysis of RNA-seq and m^1^A MeRIP-seq data from the differentially expressed genes (DEGs) was performed between sh-TRMT61A and sh-NC 5637 cells. These data were categorized into four groups: gene up-methylation and gene expression upregulation, up-methylation and gene expression downregulation, down-methylation and gene expression upregulation, and down-methylation and gene expression downregulation. A total of 834 mRNAs with Log2FC (gene expression) > 2 were identified with m^1^A down-methylation and differentially gene expression changes in transcript expression and 438 mRNAs were identified with m^1^A up-methylation and differentially gene expression changes (Figure 4A and Supplementary Table S3). Meanwhile, 497 lncRNAs were identified with m^1^A down-methylation and 277 genes were up-methylation and the percent of genes downregulated by m^1^A up-methylation was 21.6% (Figure 4B and Supplementary Table S3). Taken together, these data suggest that changes in m^1^A modifications altered gene expression.

### 3.4 m^1^A methylation affected cellular processes, biological regulation, and cancer-related pathways in BLCA

**FIGURE 5:**
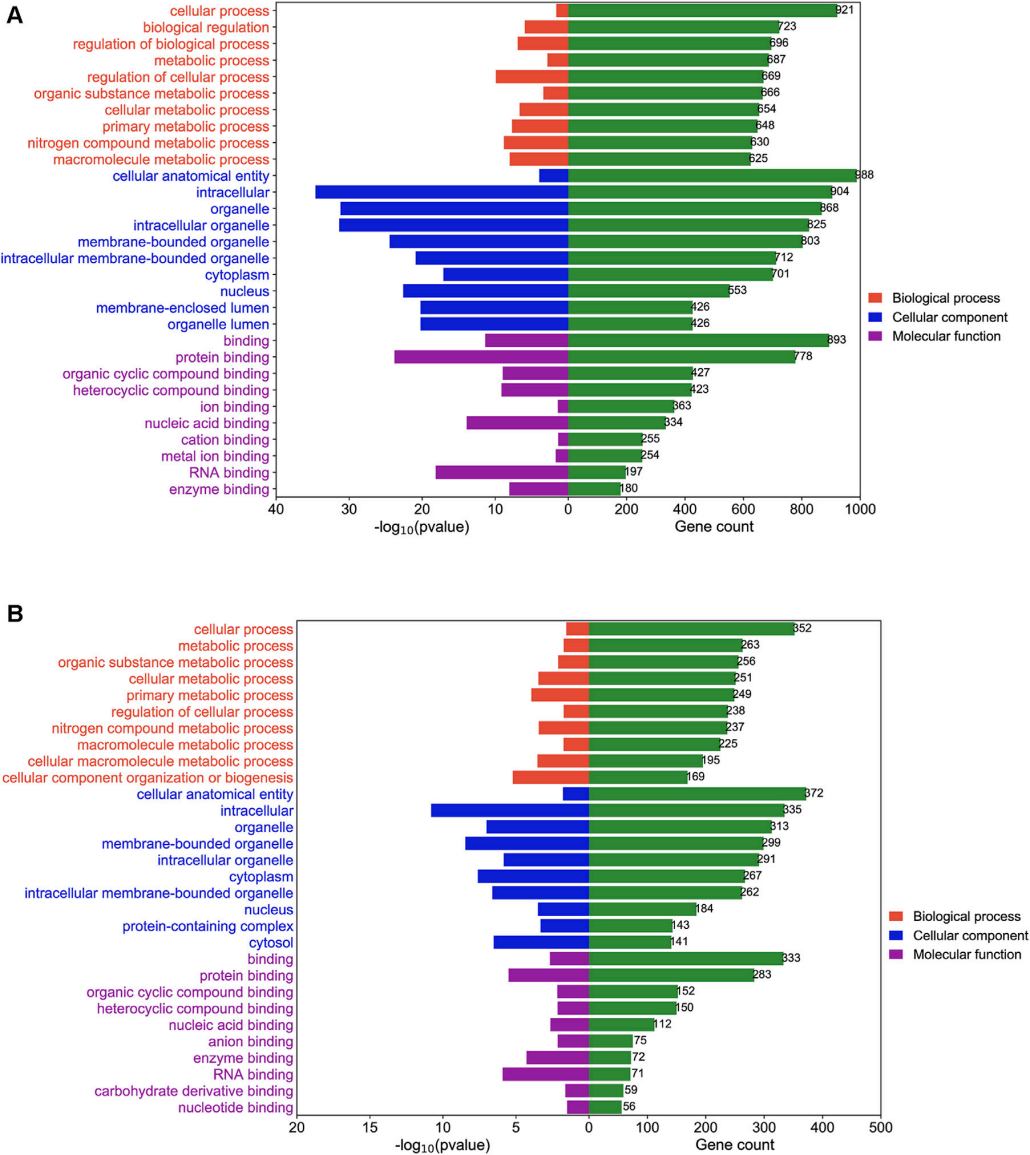
Gene Ontology (GO) enrichment analysis of N1-methyladenosine (m^1^A) modified messenger RNAs (mRNAs) and long noncoding RNAs (lncRNAs) between short hairpin-negative control (sh-NC) and sh-tRNA methyltransferase 61A (TRMT61A) 5637 cells. **(A, B)** The top 10 GO terms for biological processes, molecular functions, and cellular components are significantly enriched for mRNAs and lncRNAs with decreased m^1^A modification in bladder cancer (BLCA) cells.

To study the mechanism by which DEGs affected BLCA in sh-NC and sh-TRMT61A 5637 cells, GO enrichment and KEGG pathway analyses were performed. GO enrichment analysis was performed based on biological processes, cellular components, and molecular functions. mRNAs with decreased m^1^A methylation after TRMT61A knockdown were mostly enriched in cellular processes and binding functions, such as RNA and/or protein binding (Figure 5A and Supplementary Table S4). LncRNA transcripts with decreased m^1^A sites after TRMT61A knockdown were significantly enriched in cellular processes, and protein domain-specific binding, as well as RNA, protein, and enzyme binding (Figure 5B and Supplementary Table S4).

**FIGURE 6:**
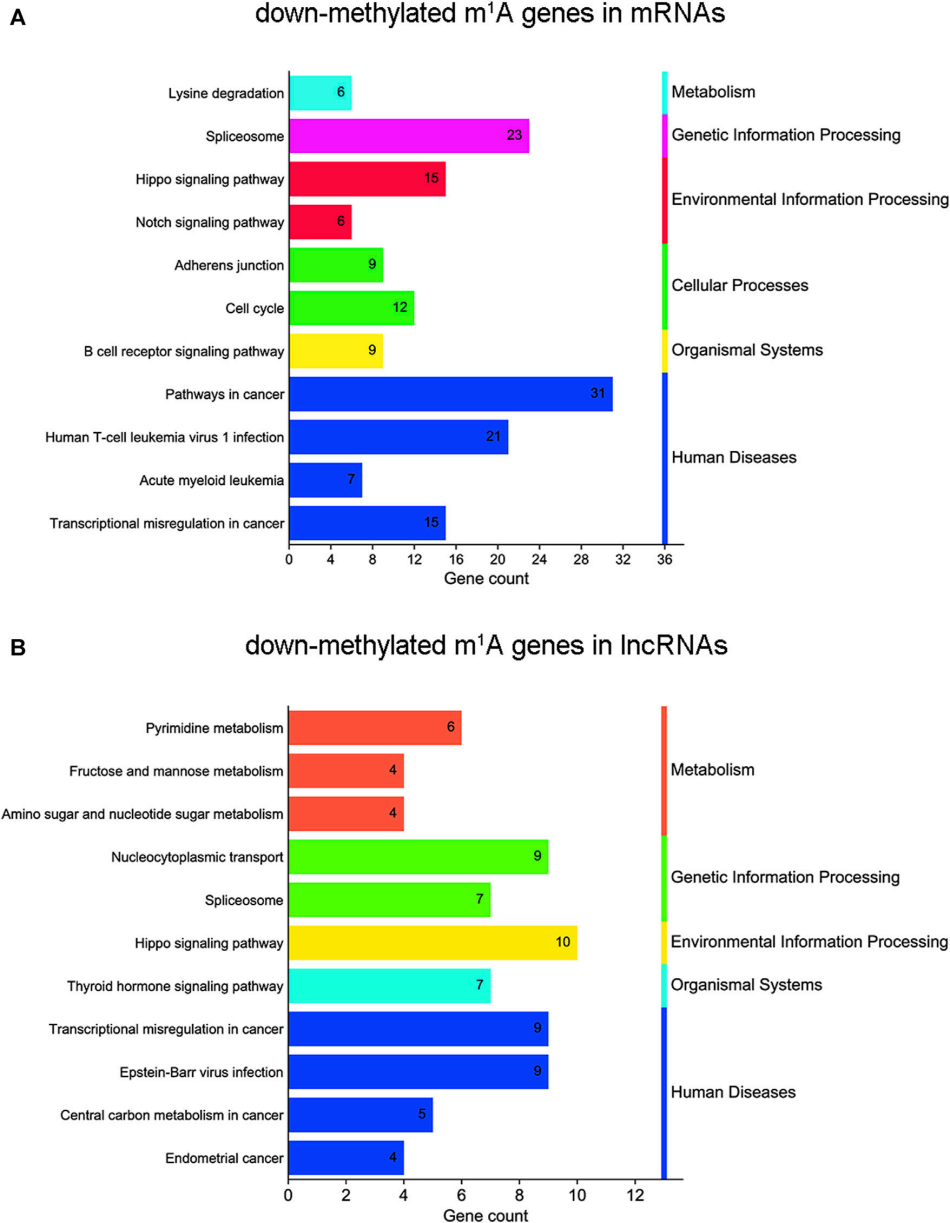
Kyoto Encyclopedia of Genes and Genomes (KEGG) pathway analysis of N1-methyladenosine (m^1^A) genes in bladder cancer (BLCA) cells’ messenger RNAs (mRNAs) and long noncoding RNAs (lncRNAs) between short hairpin-negative control (sh-NC) and sh-tRNA methyltransferase 61A (TRMT61A) 5637 cells. **(A, B)** Bar chart showing the top 11 pathways enriched by m^1^A for down-methylated mRNAs and lncRNAs.

KEGG analyses map molecular datasets from genomics, transcriptomics, proteomics, and metabolomics to explore the associated biological functions. In this study, KEGG pathway analysis revealed that m^1^A hypomethylated transcripts were significantly related to the spliceosome, transcriptional misregulation in cancer, and the Hippo signaling pathway, which were all related to cancer (Figure 6A and Supplementary Table S5). For lncRNAs, KEGG pathway analysis showed that m^1^A hypomethylated transcripts were associated with transcriptional misregulation in cancer, RNA transport, the spliceosome, and the Hippo signaling pathway (Figure 6B and Supplementary Table S5). In summary, differentially m^1^A methylated transcripts in BLCA cells were enriched in pathways involved in cellular processes, biological regulation, and cancer.

### 3.5 A COX model of 29 transcripts identified by least absolute shrinkage and selection operator (Lasso) regression predicted poor prognosis in human BLCA tissues

**FIGURE 7:**
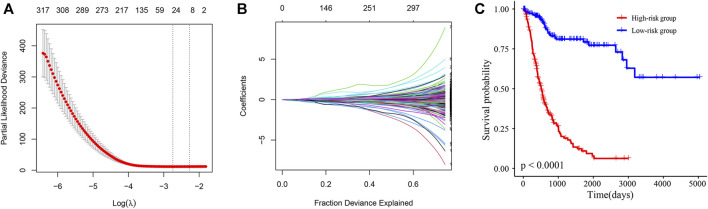
A N1-methyladenosine (m^1^A)-modified messenger RNA (mRNA) and long noncoding RNA (lncRNA) prognostic model stratifies the risk of bladder cancer (BLCA). **(A, B)** The best parameter (λ) is selected based on the least absolute shrinkage and selection operator (Lasso) model. **(C)** Kaplan-Meier survival curves of high/low-risk group patients in the Cancer Genome Atlas (TCGA) BLCA cohort. The Cox model of 29 transcripts identified by Lasso regression stratifies the risk of BLCA.

Next, Lasso-Cox regression analysis was performed on mRNAs and lncRNAs differentially expressed in sh-NC and sh-TRMT61A 5637 cells to construct a risk score for predicting overall survival. Figures 7A, B show that the best parameter (λ) is selected based on the Lasso model. According to the best λ, 29 genes (coefficients are shown in Supplementary Table S6) were considered candidate genes that distinguished patients with BLCA into high-risk and low-risk groups in the Cancer Genome Atlas (TCGA) database. The overall survival was significantly poorer in the high-risk group (*n* = 214) of patients than that in the low-risk group (*n* = 192) of patients with a risk score of 0.89 (*p* < 0.0001, Figure 7C). The patient prognosis data suggests that the expression of genes with reduced m^1^A modification after TRMT61A knockdown predicts poor prognosis in bladder cancer patients and m^1^A methylation is a potential therapeutic target.

### 3.6 Construction of a lncRNA-miRNA-mRNA competing endogenous RNA (ceRNA) network

**FIGURE 8:**
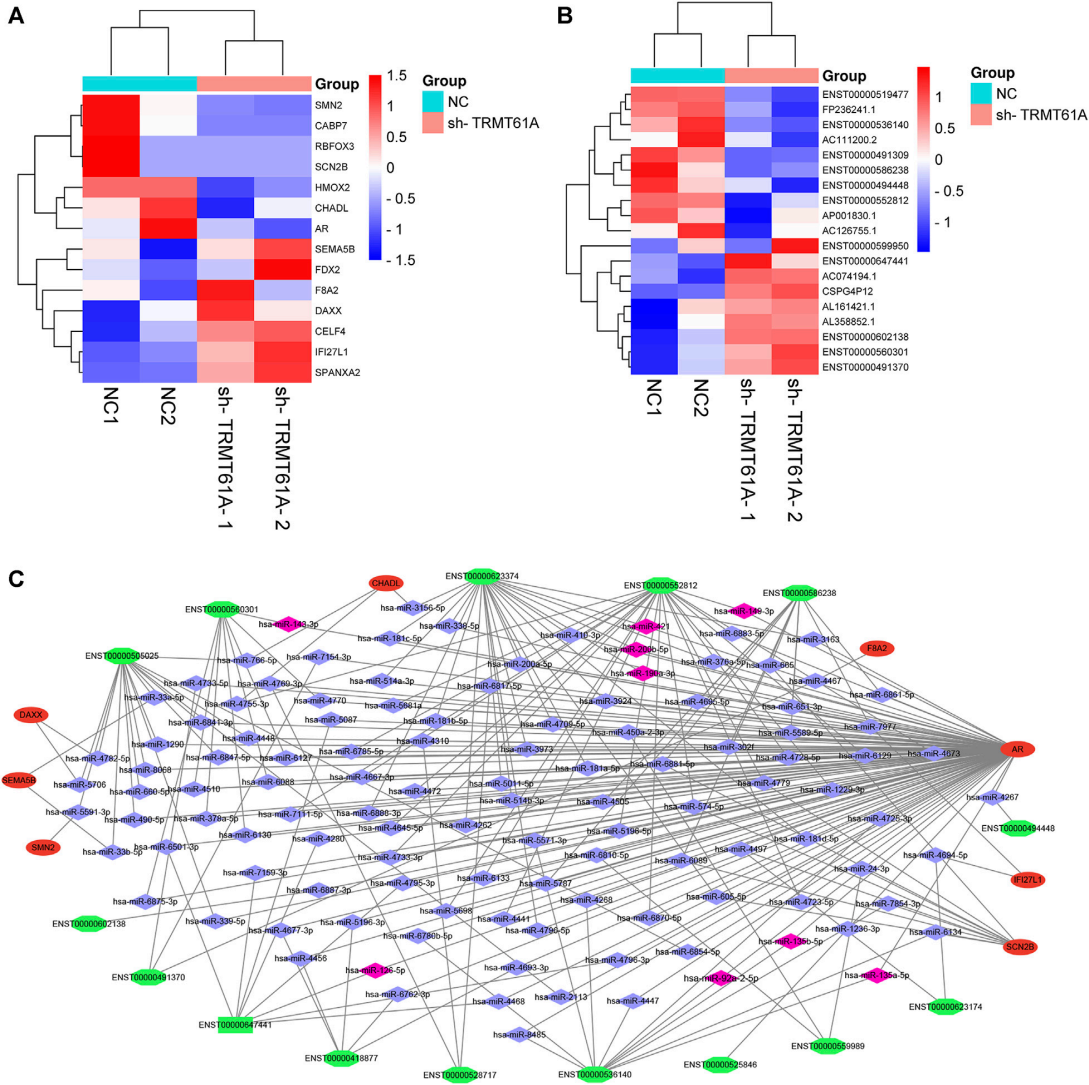
Construction of a long noncoding RNA (lncRNA)-microRNA (miRNA)-messenger RNA (mRNA) competing endogenous RNA (ceRNA) network. **(A, B)** Filtering diminished N^1^-methyladenosine (m^1^A) peaks (methylated RNA immunoprecipitation sequencing [MeRIP-Seq] data) with differentially expressed mRNA and lncRNA genes (RNA sequencing [RNA-seq] data) in short hairpin tRNA methyltransferase 61A (sh-TRMT61A) cells, compared to that of short hairpin-negative control (sh-NC) cells. **(C)** The lncRNA-miRNA-mRNA ceRNA network. Green represents lncRNA, red represents mRNA, blue represents miRNA, and pink represents reported miRNA.

We then took the intersection of all mRNAs and lncRNAs differentially methylated and differentially expressed between sh-NC and sh-TRMT61A 5637 cells, and we identified 14 mRNAs (Figure 8A) and 19 lncRNAs (Figure 8B) with different m^1^A methylation peaks and expression between sh-NC and sh-TRMT61A 5637 cells. According to ceRNA theory, endogenous RNAs competitively bind to the same miRNAs, thereby regulating mutual expression (Salmena et al., 2011). Therefore, the current study used TargetScan and StarBase to identify a lncRNA-miRNA-mRNA ceRNA network. A total of 158 lncRNA-miRNA and 3,868 miRNA-mRNA interaction pairs were identified. Finally, 118 lncRNA-miRNA-mRNA interaction pairs were revealed. The ceRNA network (Figure 8C) was constructed via Cytoscape 3.9.1, and included 15 lncRNAs (seven upregulated and eight downregulated; sh-TRMT61A 5637 cells vs*.* sh-NC 5637 cells), 8 mRNAs (four upregulated and four downregulated; sh-TRMT61A 5637 cells vs*.* sh-NC 5637 cells), and 118 miRNAs. Among these miRNAs, nine (hsa-miR-92a-2-5p, hsa-miR-135b-5p, hsa-miR-135a-5p, hsa-miR-126-5p, hsa-miR-190a-3p, hsa-miR-200b-5p, hsa-miR-421, hsa-miR-149-3p, and hsa-miR-143-3p) that regulate androgen receptor (AR) expression have been previously reported (Williams et al., 2013; Kroiss et al., 2015; Xu et al., 2015; Du et al., 2016; Meng et al., 2016; Zhai et al., 2017; Bao et al., 2020; Liu et al., 2020). Additionally, the constructed lncRNA-miRNA-mRNA ceRNA network exhibited good predictive ability.

### 3.7 Expression of candidate genes correlated with worse overall survival in patients with BLCA

**FIGURE 9:**
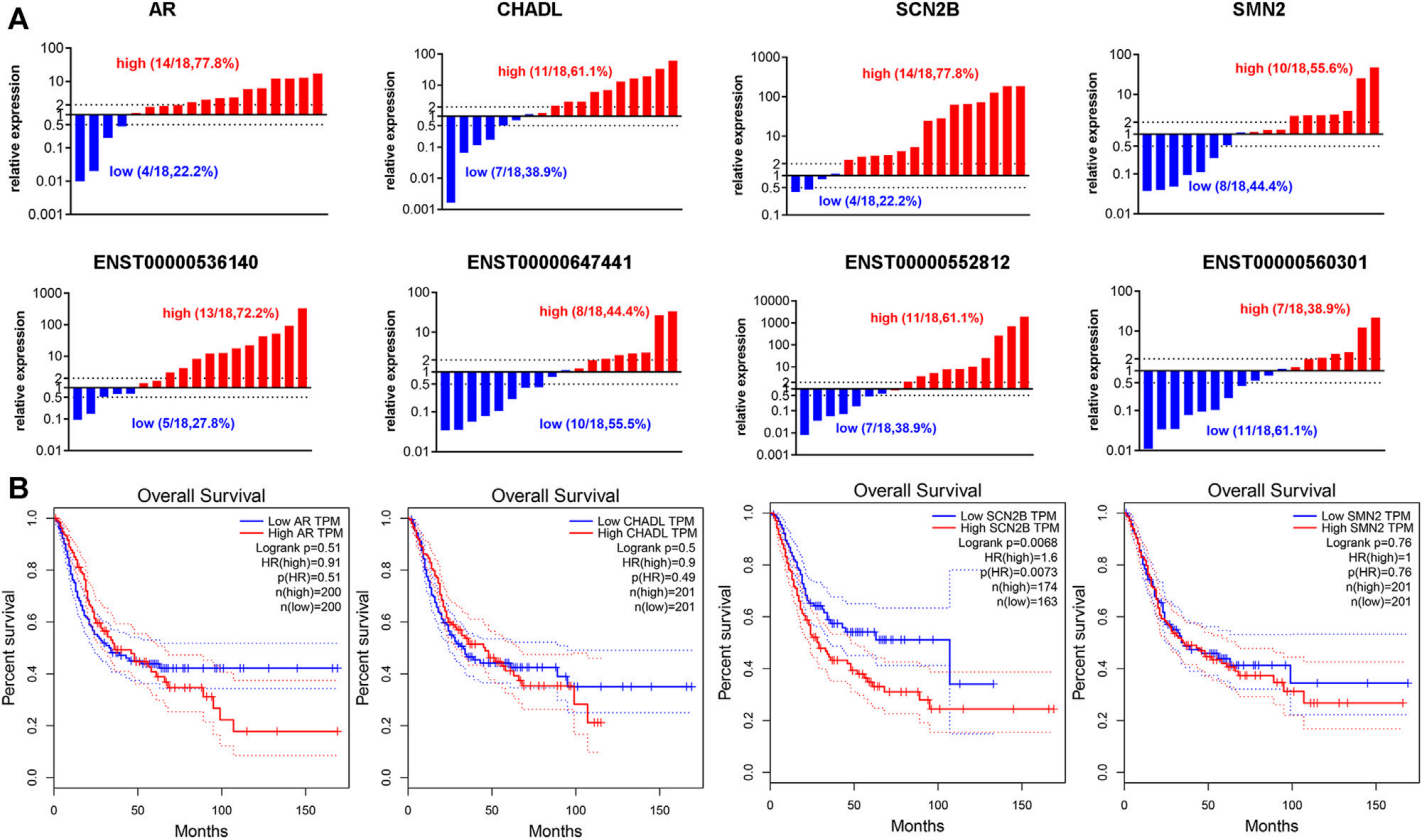
Expression of N1-methyladenosine (m^1^A)-regulated messenger RNAs (mRNAs) and long noncoding RNAs (lncRNAs) in bladder cancer (BLCA) tissues. **(A)** The RNA expression levels of androgen receptor (AR), chondroadherin-like (CHADL), sodium voltage-gated channel beta subunit 2 (SCN2B), survival of motor neuron 2 (SMN2), ENST00000536140, ENST000000647441, ENST00000552812, and ENST00000560301 in BLCA and adjacent tissues, as examined by reverse transcriptase-quantitative polymerase chain reaction (RT-qPCR) (*n* = 18). **(B)** Gene expression of AR, CHADL, SCN2B, and SMN2 in BLCA tissues extracted from the Cancer Genome Atlas (TCGA) database. Kaplan-Meier analysis examines the prognostic value of the four transcripts according to their median expression levels in BLCA tissues.

Our previous results suggested that TRMT61A is highly expressed in BLCA (Shi et al., 2015), indicating that TRMT61A functions as a cancer-promoting gene in BLCA. Therefore, the mRNA genes that were downregulated in 5637 cells after TRMT61A knockdown, including sodium voltage-gated channel beta subunit 2 (SCN2B), AR, chondroadherin-like (CHADL), and survival of motor neuron 2 (SMN2) were examined. According to the ceRNA interaction network analysis results, the related lncRNAs included ENST00000552812, ENST00000560301, ENST00000647441, and ENST00000536140. Among these lncRNAs, ENST00000552812, ENST00000560301, and ENST00000536140 retained introns of calcium voltage-gated channel auxiliary subunit beta 3, DnaJ heat shock protein family (Hsp40) member C17, and prolyl 3-hydroxylase 3 (P3H3), respectively, and ENST000000647441 was a nonsense-mediated decay transcript of D1T1. In addition, 18 pairs of collected BLCA specimens were analyzed and indicated that AR, SCN2B, and ENST00000536140 expression were elevated in BLCA tissues compared to that in normal tissue controls (Figure 9A). However, the lncRNAs analyzed in this study were new lncRNAs, and the prognostic value of these lncRNAs has yet to be determined. Therefore, the four mRNAs that had prognostic value for patients with BLCA were examined and survival analysis was performed based on the expression of the eight m^1^A methylation-driven mRNAs from the TCGA database. As shown in Figure 9B, among the potential transcripts, high levels of SCN2B in tumor tissues predict poor survival in patients with BLCA (*p* = 0.0073, log-rank test), suggesting that m^1^A-regulated mRNAs may be used as biomarkers in BLCA survival prediction.

## 4 Discussion

The m^1^A RNA modification reportedly plays a pivotal role in various functional activities and has recently drawn increasing research attention. Many bioinformatics analyses have suggested that m^1^A-regulating transcripts play essential roles in various cancers (Zhao et al., 2019; Wang et al., 2020; Li et al., 2021; Zheng et al., 2021; Jin et al., 2022; Mao et al., 2022). However, the effects of m^1^A on BLCA remain poorly understood. In this study, the features and patterns of RNA m^1^A modifications in BLCA were profiled, including the extent of m^1^A modification, m^1^A distribution in mRNAs and lncRNAs, and consensus m^1^A methylation motifs.

Currently, MeRIP-seq is used to study the distribution sites and expression levels of m^1^A on transcripts in mammalian cells. Few studies on the role of m^1^A modification in mRNAs existed until those by Dan et al. (Dominissini et al., 2016) and Li et al. (Li et al., 2017), who provided a transcriptome-wide mapping of m^1^A in HEK293T cells. In contrast to m^6^A, the distribution of m^1^A in mRNAs is unique in its proximity to the start codon, a pattern distinct from the 3′UTR enrichment of m^6^A (Meyer et al., 2012). The current m^1^A sequencing results identified m^1^A modifications around the 5′UTR, the start codon, and CDS. Additionally, the m^1^A modification in 5637 BLCA cells mainly occurs in the CAGGC motif, whereas Li et al. (Li et al., 2017) report a GUUCRA motif. Moreover, most of the methylated sequences within mRNA and lncRNAs in BLCA cells contained one m^1^A peak, whereas a few of them contained three or more sites. In addition, differentially methylated genes after TRMT61A knockdown were detected and shown to be involved in many important biological pathways, such as cancer pathways, the Hippo signaling pathway, transcriptional misregulation in cancer, and the AMP-activated protein kinase (AMPK) signaling pathway. Previous studies have reported that AMPK contributes to the aberrant activation of metabolic pathways, mitochondrial dynamics and functions, and epigenetic regulation, which are hallmarks of cancer; targeting AMPK may open up a new avenue for cancer therapies (Hsu et al., 2022).

Many studies have reported that methylation of mRNAs and lncRNAs regulates numerous processes affecting cancer cell function by enhancing RNA stability, increasing RNA nuclear accumulation, and promoting the decay of some RNAs (Jin et al., 2022). Recent studies have shown that m^1^A-regulated transcripts modulate phosphoinositide 3-kinase/protein kinase B/mechanistic target of rapamycin kinase and ErbB in gastrointestinal cancer (Zhao et al., 2019) and that m^1^A modifications play a role in tumorigenesis by affecting mRNA stability (Macari et al., 2016; Wu et al., 2022). In addition, analysis of RNA-seq data and clinical information from the TCGA database of head and neck squamous cell carcinoma samples has revealed that the expression of m^6^A/m^5^C/m^1^A-related lncRNAs is associated with patient prognosis and the immune microenvironment (Wang E. et al., 2021).

A combined analysis of the current RNA-seq and MeRIP-seq data and predictive models according to the results of the Lasso Cox regression were constructed, and 29 transcripts predicted poor prognosis in human BLCA. Simultaneously, a lncRNA-miRNA-mRNA network was constructed based on lncRNA-miRNA and miRNA-mRNA interaction pairs, and a potential mRNA-miRNA-lncRNA-ceRNA triplet network associated with BLCA prognosis was constructed. Among the predicted miRNAs, nine have been confirmed to be involved in the regulation of AR expression (Williams et al., 2013; Kroiss et al., 2015; Xu et al., 2015; Du et al., 2016; Meng et al., 2016; Zhai et al., 2017; Bao et al., 2020; Liu et al., 2020), demonstrating the strong reliability of the predicted miRNA.

The m^1^A-modified transcripts SCN2B and ENST000000536140 were highly expressed in BLCA tissues. SCN2B is a novel adhesion molecule that promotes the migration of human breast cancer cells (Chioni et al., 2009). Additionally, ENST000000536140 is a retained intron of P3H3 and is a new target for epigenetic silencing in breast cancer (Shah et al., 2009); however, its function in BLCA is unclear. Therefore, SCN2B and ENST00000536140 may play a role in BLCA development. Among the miRNAs predicted to bind to ENST000536140 and SCN2B, two miRNAs, hsa-miR-6134 and hsa-miR-7854-3p, bind to both transcripts. Has-miR-6134 has been reported to be involved in liver cancer and lung cancer (Mandal et al., 2021; Chakraborty and Nath, 2022). These data indicate that m^1^A methylation participates in cancer progression by modulating cancer-related mRNAs, lncRNAs, and miRNAs. However, further experiments are required to confirm these findings.

In summary, the genome-wide integrated analysis of RNA m^1^A methylation and the transcriptome identified m^1^A-regulated mRNAs and lncRNAs and characterized the crosstalk between mRNA and lncRNA m^1^A methylation and expression. Additionally, a methylation-driven lncRNA-based signature with potential clinical applications for predicting the prognosis of BLCA was identified.

## Data Availability

All datasets generated for this study are included in the manuscript and the Supplementary Files. The publicly available datasets analyzed in the current study are available in TCGA (https://www.cancer.gov/ccg/research/genome-sequencing/tcga) and GEPIA2 (http://gepia2.cancer-pku.cn/#index). The datasets presented in this study can be found in online repositories (GSE255629, https://www.ncbi.nlm.nih.gov/geo/query/acc.cgi?acc=GSE255629).

## Abbreviations

AR, androgen receptor; BLCA, bladder cancer; CDS, coding sequence; CHADL, chondroadherin like; DEGs, differentially expressed genes; GO, Gene Ontology; KEGG, Kyoto Encyclopedia of Genes and Genomes; lncRNA, long non-coding RNA; mRNAs, messenger RNAs; m^6^A, N^6^-methyladenosine; m^1^A, N^1^-methyladenosine; m^7^G, N^7^-methylguanosine; m^5^C, 5-methylcytosine; MIBC, muscle-invasive bladder cancer; MeRIP-seq, methylated RNA immunoprecipitation sequencing; NMIBC, non-muscle-invasive bladder cancer; RT-qPCR, reverse transcriptase-quantitative polymerase chain reaction; RNA-seq, RNA sequencing; SCN2B, sodium voltage-gated channel beta subunit 2; SMN2, survival of motor neuron 2; TRMT61A, tRNA methyltransferase 61A; TRMT10C, tRNA methyltransferase 10C; UHPLC, ultra-high-performance liquid chromatography; 5′ UTRs, 5′-untranslated regions.
