# Supplementary figures and images for: Transcriptome-wide 1-methyladenosine functional profiling of messenger RNA and long non-coding RNA in bladder cancer
Source: Front Genet. 2024 Feb 28;15:1333931. doi: 10.3389/fgene.2024.1333931 (PMC10933092; doi:10.3389/fgene.2024.1333931)

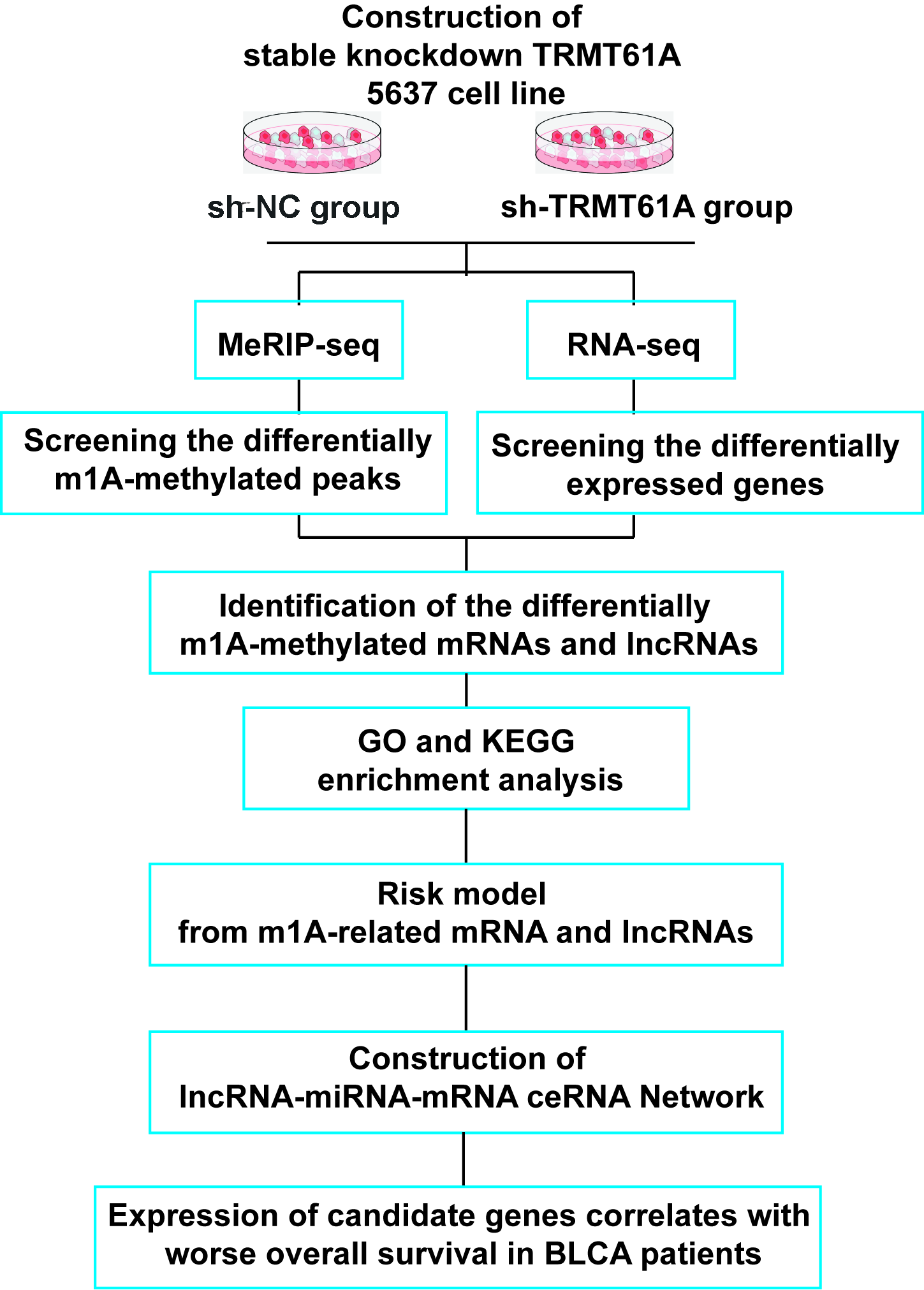

Supplement: Supplementary file 3 [file Image1.TIF]
