# Supplementary material for: Transcriptome-wide 1-methyladenosine functional profiling of messenger RNA and long non-coding RNA in bladder cancer
Source: Front Genet. 2024 Feb 28;15:1333931. doi: 10.3389/fgene.2024.1333931 (PMC10933092; doi:10.3389/fgene.2024.1333931)
Supplement: Supplementary file 8 [file DataSheet2.docx]

load("~/R_data/TCGA-BLCA-Allsample(T-N)-expr-pdata.Rdata")

load("~/R_data/blca_svdata.Rdata")

mrna.up<-read.csv("./mRNAs up.csv",header =T)

mrna.down<-read.csv("./mRNAs down.csv",header =T)

lncrna.up<-read.csv("./LncRNAs up.csv",header =T)

lncrna.up<-lncrna.up[!duplicated(lncrna.up$GeneName),]

lncrna.down<-read.csv("./LncRNAs down.csv",header =T)

lncrna.down<-lncrna.down[!duplicated(lncrna.down$GeneName),]

lasso.genes<-c(mrna.up$gene_name,mrna.down$gene_name,lncrna.up$GeneName,lncrna.down$GeneName)

lasso.genes<-lasso.genes[!duplicated(lasso.genes)]

svdata<-svdata[(rownames(svdata)%in%colnames(exp)),]

svdata<-svdata[!is.na(svdata$OS.time),]

svdata<-svdata[svdata$OS!=0|svdata$OS.time!=0,]

exp<-exp[,rownames(svdata)]

table(lasso.genes %in% rownames(exp) )

lasso.genes<-lasso.genes[lasso.genes %in% rownames(exp)]

library(survival)

library(survminer)

library(glmnet)

exp<-exp[lasso.genes,]

exp[1:5,1:5]

surv.obj <- data.matrix(Surv(svdata$OS.time, svdata$OS))

fit = glmnet(x = t(as.matrix(exp)),

y = surv.obj,

family = "cox", alpha = 1, lambda = NULL)

#pdf("A_lasso.pdf", width = 5, height = 5)

plot(fit, xvar = "dev", label = TRUE)

cvfit = cv.glmnet(x = t(as.matrix(exp)),

y = surv.obj,

nfold=10,

family = "cox", type.measure = "class")

plot(cvfit)

cvfit$lambda.min

myCoefs <- coef(cvfit, s="lambda.min");

lasso_fea <- myCoefs@Dimnames[[1]][which(myCoefs != 0 )]

surv.dat<-cbind(svdata[,26:27],as.data.frame(t(exp[lasso_fea,])))

cox <- coxph(Surv(OS.time, OS) ~ ., data = surv.dat)

riskScore <- predict(cox,type="risk",newdata=surv.dat)

sv.input<-cbind(svdata[,26:27],riskScore)

res.cut <- surv_cutpoint(sv.input, time = "OS.time",

event = "OS",

variables = names(sv.input)[3],

minprop = 0.3)

res.cat <- surv_categorize(res.cut)

if(T){

fit<- survfit(Surv(OS.time, OS) ~ riskScore, data = res.cat)

ggsurvplot(fit, pval = TRUE,linetype = c("solid", "solid"),

palette = c("red","blue"),

legend.title="",legend=c(0.7,0.9),legend.labs=c("High-expression","low-expression"),

conf.int = F)

}
