# Supplementary material for: Transcriptome-wide 1-methyladenosine functional profiling of messenger RNA and long non-coding RNA in bladder cancer
Source: Front Genet. 2024 Feb 28;15:1333931. doi: 10.3389/fgene.2024.1333931 (PMC10933092; doi:10.3389/fgene.2024.1333931)

**Western blot**

Figure 1B

**GAPDH**


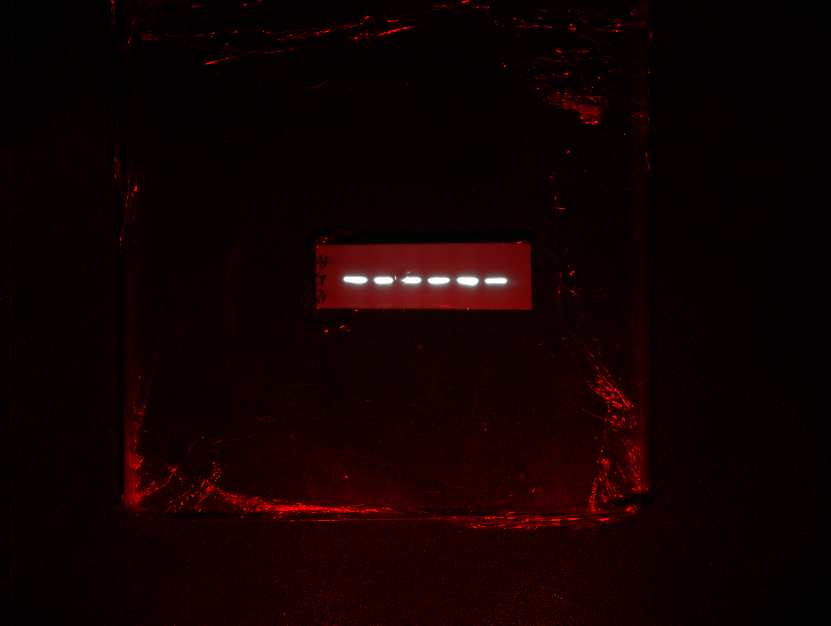


**sh-TRMT61A**

**sh-NC**

**37 KD**

**34 KD**

**43 KD**


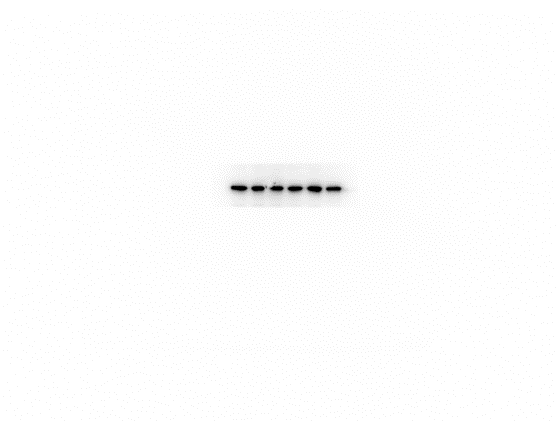


**TRMT61A**


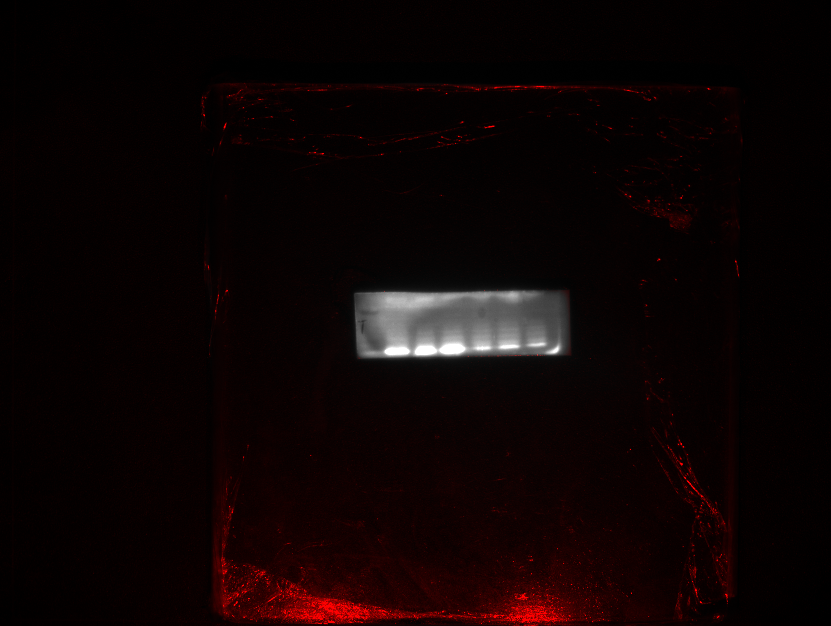


**sh-TRMT61A**

**sh-NC**

**34 KD**

**43 KD**


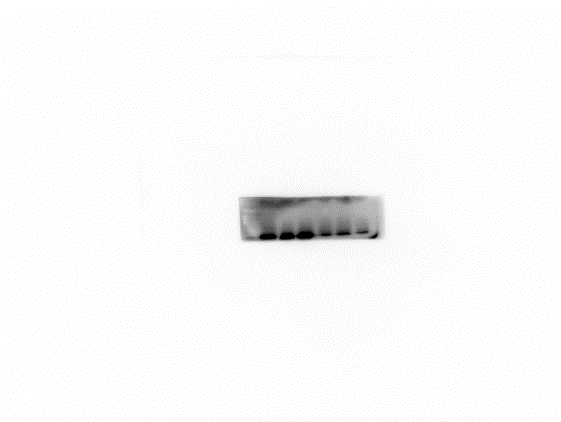


**Figure 1C**

**m^1^A dot blot MB (Methylene blue)**


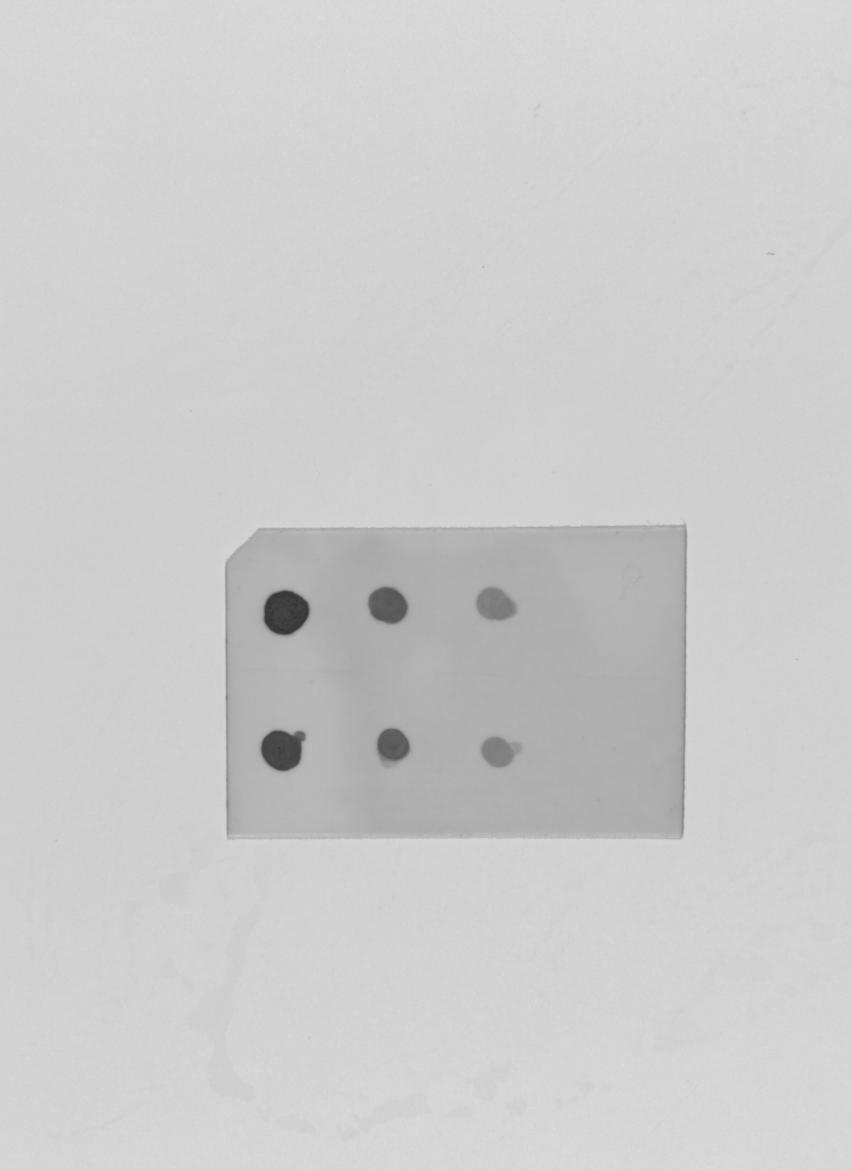

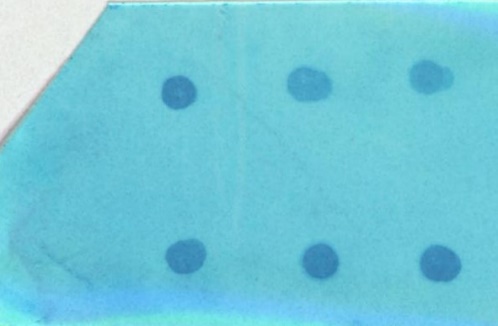

Supplement: Supplementary file 10 [file DataSheet1.docx]
